# Challenges and Strategies of Laboratory Diagnosis for Newly Emerging Influenza Viruses in Taiwan: A Decade after SARS

**DOI:** 10.1155/2015/805306

**Published:** 2015-07-28

**Authors:** Jih-Hui Lin, Ho-Sheng Wu

**Affiliations:** ^1^National Influenza Center, Centers for Disease Control, No. 161, Kung-Yang Street, Taipei 11513, Taiwan; ^2^School of Medical Laboratory Science and Biotechnology, Taipei Medical University, 250 Wu-Hsing Street, Taipei 11065, Taiwan

## Abstract

Since the first case of severe acute respiratory syndrome (SARS) in Taiwan was identified in March 2003, viral respiratory infections, in particular the influenza virus, have become a national public health concern. Taiwan would face a serious threat of public health problems if another SARS epidemic overlapped with a flu outbreak. After SARS, the Taiwan Centers for Disease Control accelerated and strengthened domestic research on influenza and expanded the exchange of information with international counterparts. The capacity of influenza A to cross species barriers presents a potential threat to human health. Given the mutations of avian flu viruses such as H7N9, H6N1, and H10N8, all countries, including Taiwan, must equip themselves to face a possible epidemic or pandemic. Such preparedness requires global collaboration.

## 1. Introduction

The growing interconnectedness and complexity of the world presents an increasing challenge to influenza prevention and control. Distinct seasonality of human influenza was observed in both the Northern and Southern Hemispheres. The rates and severity of annual epidemics vary from year to year and place to place, depending on the strain and subtype circulating and susceptibility of the population. The emergence of pandemic influenza H1N1 from a swine origin in 2009 [1], the sporadic zoonotic cases of swine origin influenza viruses (SOIV) in 2011-2012 [2], and avian influenza A virus subtypes, including H5N1, H7N7, H9N2, H7N3, and H7N9, were all experienced to infect humans during the past years. All of these indicate the importance of rapid laboratorial diagnosis of influenza virus and early detection of epidemics.

Influenza virus is one of the major pathogens for both human and animals; it consists of eight single-stranded RNA gene segments, and the viral particle has two major glycoproteins on its surface: hemagglutinin and neuraminidase. Based on different surface glycoproteins, influenza A viruses are further classified into 18 different HA antigens (H1–H18) and 11 different NA antigens (N1–N11). The subtype H16 influenza A virus was isolated from black-headed gulls from Sweden and Norway in 2004 [3]. H17 was discovered in 2012 in fruit bats [4], and the most recent H18 was discovered in a Peruvian bat in 2013 [5]. With the 18 different hemagglutinin and 11 different neuraminidase subtypes, different combinations of these proteins are considerable antigenic variation among influenza viruses. Almost all HA (H1–H16) and NA (N1–N9) subtypes of influenza viruses are found in aquatic birds, but those subtypes circulating in humans are limited to three HA (H1, H2, and H3) and two NA (N1 and N2) subtypes before 1997 when the first outbreak of avian influenza A (H5N1) virus in humans occurred in Hong Kong.

The specific structure of influenza virus allows genetic reassortment and results in changes in the surface glycoproteins of the virus and usually causes devastating epidemics and pandemics in humans, birds, pigs, horses, and seals [6]. Scientists keep studying the molecular biology of the virus and using reverse genetics methods in generating influenza viruses for better understanding of them but still cannot realize why some strains are transmitted well and cause pandemics; likewise, they cannot find out why the viruses lead to disease in one species and not in another.

**Figure 1:**
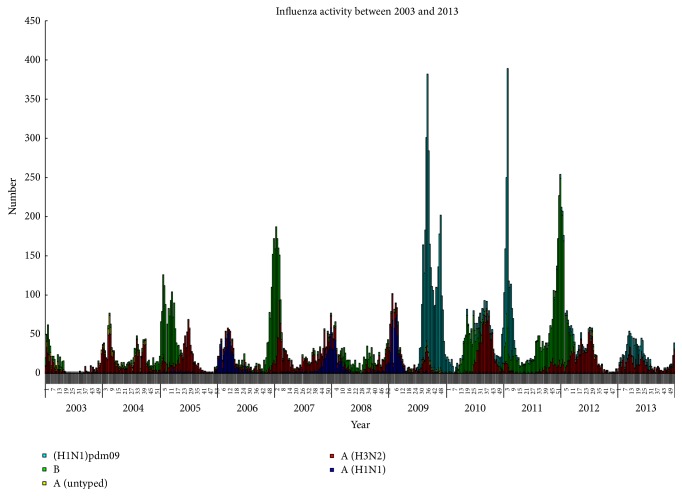
Weekly distribution of Taiwan influenza isolates, 2003–2013. Weekly distribution of Taiwanese influenza isolates based on cell culture and immunofluorescence results from January 2003 to December 2013. Different types and subtypes were colored as follows: H1N1 in indigo, pdmH1N1 in light blue, H3N2 in red, and B in green.

Influenza A virus is one of the most active pathogens in Taiwan causing regular, yearly islandwide epidemics and associated with significant morbidity and mortality in humans [7, 8]. Influenza subtypes H1N1 and H3N2 are currently the major circulating subtypes and have been infecting the human population for several decades. The molecular epidemiology of the influenza viruses circulating in Asia, including Taiwan, is already of wide international concern because strong travel makes the viruses easy to spread between Asia and other countries [9]. But some phylogeographic analyses have suggested that Southeast Asia may not act as a source of sustained annual H3N2 influenza epidemics and persistent circulation of the viruses in tropical Asian regions may be dependent on viral input from temperate regions [10]. Nevertheless, the changing patterns of influenza viruses in Taiwan revealed a dynamic interaction between subtypes; for example, the H1N1 epidemics occurred during winter periods in Taiwan and the H3N2 epidemics often appeared as two separate peaks in summer and winter reflecting the circulation of at least two different H3N2 viruses during that period (Figure 1). Data from Taiwan CDC Lin et al. indicate that H3 summer isolates were genetically and antigenically distinct from preceding seasons but were similar to viruses isolated during the following winter [8]. This observation is consistent with the summer epidemic strains being the progenitors of those dominant in the following flu season. Taiwan is located in a subtropical area of Southeast Asia with an extremely high population density in most urban areas, particularly the capital city of Taipei. Therefore, respiratory infections, especially influenza, have a public health priority after the SARS outbreaks in Taiwan.

## 2. Influenza Epidemics and Pandemics

There were 3 pandemics of influenza A viruses in the last century: the 1918 H1N1 subtype, 1957 H2N2, and 1968 H3N2 [6, 11]. Surveillance of influenza activity in Taiwan for any potential outbreak or pandemic is based on laboratory isolation of influenza viruses and sentinel general practitioner reports of influenza-like illness. It was approximately 62.7% of influenza A (H1N1 and H3N2) and 37.3% of influenza B viruses from Taiwanese isolates analyzed using postinfection ferret antisera during the past decade. Five influenza seasons were dominated by H1N1, which were preceded by H3N2 during the most recent 5 years. In addition, H3N2 viruses frequently cocirculated with influenza B viruses in several seasons (2000-01, 2004-05, and 2006-07 seasons) [8]. This illustrates that the influenza A viruses were somewhat more active in Taiwan.

Furthermore, the epidemic of human H5N1 in Hong Kong in 1997 [12] and the finding of H9N2 human isolates in China and Hong Kong in 1999 [13, 14] imply that influenza A viruses are widespread in Asia. Under these potential public health threats, Taiwan Centers for Disease Control (Taiwan CDC) coordinated monitoring of influenza activity by establishing sensitive, representative, and integrated surveillance systems. The incentive to reinforce infectious diseases surveillance system in Taiwan was that since the severe outbreak of SARS in 2003 the surveillance is performed by sentinel primary care physicians and is based on integrated clinical and virological surveillance components [15]. In addition to the sentinel system, Taiwan CDC has initiated a nationwide laboratory-based virological diseases surveillance system to collect clinical specimens for isolation and identification of influenza viruses. According to the Communicable Disease Control Act of Taiwan, all suspected influenza complicated cases need to be reported and specimens must be collected and sent to Taiwan CDC through National Notifiable Disease Surveillance System (NNDSS). Respiratory secretions from throat swabs, nasopharyngeal aspirates, or bronchoalveolar lavages were collected from patients who were suspected to have influenza infections. The diagnosis of influenza infection was confirmed by viral culture, immunofluorescence antibody stain, reverse transcriptase-polymerase chain reaction (RT-PCR), and/or a commercially available rapid diagnostic test. Currently, there are 8 clinical virology laboratories, geographically distributed in northern, central, southern, and eastern Taiwan, joining the network of Taiwan CDC Collaborating Laboratory of Virology (TCCLV) [16]. All the laboratories have to pass the proficiency tests of viral diagnosis (organized by Taiwan CDC) every year, and data were collected weekly for rapid influenza activity data analysis. More comprehensive results of surveillance data analysis will be sent to WHO collaborating centers and essential regulatory laboratories to assist in selecting virus strains for the recommendations on the composition of influenza virus vaccines.

## 3. Establishment of Taiwan National Influenza Center

The Taiwan National Influenza Center (Taiwan NIC) was established in Taipei on July 5, 2006, a special date to be chosen to remember that Taiwan was removed from the WHO's list of areas that had been affected by SARS for three years. The WHO Global Influenza Surveillance Network was established in 1952. It comprises 5 WHO collaborating centers (WHO CCs) on influenza and 112 institutions in 83 countries, which are recognized by WHO as WHO National Influenza Centers (NICs) [17]. Although Taiwan NIC has not been recognized as a member of the WHO's network of influenza centers, Taiwan has regularly volunteered to send Taiwanese influenza isolates to the WHO CCs since 1979, making it part of the international flu virus supervision network.

During the post-SARS period, Taiwan CDC speeds up and strengthens domestic research on influenza and expands the exchange of information with our international counterparts, because Taiwan would face a great threat if another SARS epidemic and flu outbreak overlapped. Given the mutations of avian flu viruses, Taiwan CDC must fully equip itself to face any possible epidemic or pandemic. Thus, laboratory diagnostic technologies were established during this period, including multiplex RT-PCR/PCR, multiplex suspension beads array, microarray, loop-mediated isothermal amplification (LAMP), and high throughput sequencing. The diagnostic capacity of Taiwan CDC would be able to handle suspected clinical specimen around 1,500 cases in a day at maximum.

## 4. Development of Laboratory Diagnostic Systems for Influenza

Emerging known and unknown influenza viruses create profound threats to public health; platforms for rapid detection and characterization of influenza viruses are critically needed to prevent and respond to any potential outbreaks in Taiwan. During the past decade, quickly developing and far-reaching technology of biotechnology were wildly applied; the most commonly used methods to identify and quantify influenza infection at Taiwan CDC can be subdivided into three broader phases, phase 1, pre-SARS, and techniques measuring viral infectivity (viral plaque assay, TCID_50_, and immunofluorescence assay); traditionally cell culture based on virus isolation has been regarded as “golden standard” for the detection and diagnosis of virus infection, and it is the technique to which all other test methods have been compared. After SARS outbreak, the laboratory diagnostic technics were moved to the next phase, which are to examine viral nucleic acid and protein (qPCR, immunoblotting, ELISA, and hemagglutination assay). The development of molecular methods for the direct identification of specific viral genome from clinical sample is one of the greatest achievements during this period. The advance and current phase of the Center for Research, Diagnostics and Vaccine Development of Taiwan CDC is to measure the expression levels of large numbers of genes simultaneously or to genotype multiple regions of a genome (DNA microarray, liquid chip array, and SNP). Clearly nucleic acid amplification techniques including PCR, nucleic acid sequence-based amplification, and multiplex detection system are proven technologies for rapid detection and molecular identification for most known human influenza viruses. Influenza viruses are traditionally detected using specific antibody-based immunoassays or immune-fluorescence assays. On the other hand, RT-PCR and real-time RT-PCR assays using specific primers against vial nucleic acids were more advanced and specific and provide faster results than end-point assays of influenza detection and in many cases have sensitivities equal to or better than cell culture. Unfortunately, high mutation rates of influenza virus may lead to extensive changes in viral nucleic acid sequences making dedicated PCR primer use irrelevant; therefore, it is highly demanded to develop rapid and universal identification and detection technologies. Furthermore, facing the growing threat of interspecies transmission of influenza viruses resulting in the emergence of new infectious pathogens, the DNA microarray was applied in Taiwan CDC for diagnosis, identification, sequencing, and subtyping of influenza virus. Recently, a new molecular biology-based microbial detection method for rapid identification of multiple virus types in one specimen has been developed. Microbial Detection Array (MDA) detects viruses using probes against genomic DNA sequence within 24 hours. Each probe tests for a particular sequence of DNA and small groups of probes can be used to check for specific viruses up to the species level, different from current PCR technologies that focus on small, prioritized sets of high-risk biological pathogens. MDA, however, can identify a broad range of organisms, including pathogens on a priority screening list, sequenced bacteria, or viruses that might not be anticipated. Such technology has great potential for improving diagnostic processes and in different applications. New technologies have been continuously added in the field of biomedical sciences, which has gradually enriched science and enormously improved the quality and quantity of research output and will provide better preparedness for the next outbreak from a new emerging influenza virus.

## 5. Emergence of Novel Pandemic Influenza Viruses

**Figure 2:**
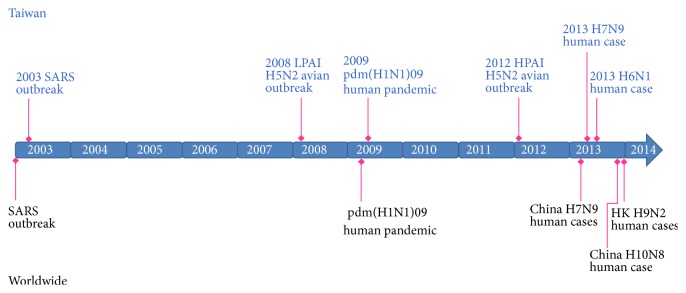
Epidemic events of influenza A virus in Taiwan, 2003–2013. Shown are the events in Taiwan (top) in the context of the timeline of the influenza epidemics worldwide (bottom).

There were several zoonotic influenza strains that infected poultry and human during the past decade in Taiwan (Figure 2). In April 2009, a new strain of H1N1 from Mexico was found to be a novel strain of influenza for which current vaccines against seasonal flu provided little protection. This new H1N1 strain resulted from a reassortment of bird, swine, and human flu viruses [18]. In June 2009, the WHO declared the outbreak of a pandemic [19] which was named as pandemic H1N1/09 virus (pdmH1N1) in July 2009 [20]. It is possible that the virus has been circulating in human population since some time in the past and had not been detected.

The first case of H1N1 detected in Taiwan was confirmed on May 20, 2009, and another 9 positive cases in a row were identified within a week. The pdmH1N1 virus was isolated in late May 2009, causing a community outbreak in early July and then spreading islandwide [21]. Taiwan CDC provided free seasonal influenza vaccine started from October; people received their vaccination at over 3,500 contracted hospitals and clinics. Then the Taiwan government approved an inactivated vaccine with influenza A/California/7/2009 (H1N1) strain known as AdimFlu-S (Adimmune Corporation, Taichung, Taiwan) and initiated mass vaccinations in November [22]. Free AdimFlu-S vaccines were provided giving the priority to schoolchildren, the elderly, and front-line healthcare personnel. Due to the delay in vaccine development and delivery in 2010, health authorities used both vaccination and school closure to control pdmH1N1 [22–24]. Overall, the vaccine coverage rates were 76.9% for children and 24.6% for civilians in late July 2010 [22, 24, 25].

Confirmed case of pdmH1N1 influenza virus infection in Taiwan is defined as an individual with laboratory-confirmed pdmH1N1 influenza virus infection by one or more of the following tests: (1) real-time reverse transcriptase-polymerase chain reaction (RT-PCR), viral culture, or fourfold rise in pdmH1N1 influenza virus-specific neutralizing antibodies [26]; (2) viral culture and (3) RT-PCR which can reliably identify the presence of pdmH1N1 influenza virus in specimens, especially RT-PCR, which has the highest sensitivity and specificity. With RT-PCR, pdmH1N1 influenza virus will test positive for influenza A and negative for seasonal H1 or H3. Meanwhile, commercially available rapid influenza diagnostic tests (RIDTs) detect influenza viral nucleoprotein antigen and are capable of providing results within 30 minutes. The sensitivity of RIDTs for detecting pdmH1N1 influenza varied from 10% to 70% and is directly related to the amount of virus in the specimen but inversely related to the threshold cycle value of the test [27]. Rapid tests for influenza may detect the antigen from either influenza A or B in respiratory specimens with a high specificity (>95%), but the negative result from a rapid test does not rule out influenza infection, and most of rapid tests cannot distinguish pdmH1N1 from H3N2 influenza A viruses [21].

The oseltamivir-resistant pandemic influenza A (H1N1) virus strain in Taiwan was first isolated from a 20-year-old male in October 2009. The H1N1 virus isolated before the patient received oseltamivir treatment was sensitive to oseltamivir. However, three days after initiation of treatment, the virus isolated from the same patient shows a mutation involving the substitution of a histidine for tyrosine at position 275 (H275Y), which is resistant to oseltamivir. Oseltamivir treatment was not associated with statistically significant reduction in the duration of viral shedding, thereby proving ineffective in preventing viral spread in community. The role of swine in the genesis of this pandemic was again apparent. The lesson learned is that the pandemics of influenza can arise anywhere in the world and that global surveillance is merited.

## 6. Imported Human H7N9 Infections

Rare cases of humans infection with avian influenza A viruses have been reported, with the exception of highly pathogenic avian influenza A (H5N1) viruses, which have caused 667 infections and 393 deaths as of June 27, 2014 [28]. In 2003, 89 people were infected with influenza A virus of H7N7 subtype that caused conjunctivitis and one fatality in Netherlands [29]. The recent sporadic infections of humans in China with a previously unrecognized avian influenza A virus of the H7N9 subtype A have caused concern owing to the appreciable case fatality rate associated with these infections (more than 25%), potential instances of human-to-human transmission [30], and the lack of preexisting immunity among humans to viruses of this subtype. Avian influenza A (H7N9) is a subtype of influenza viruses that have been detected in birds in the past. This particular H7N9 virus had not previously been seen in either animals or people until it was found in March 2013 in China [31]. Since then, a total of 450 cases with 165 deaths have been reported, and infections in both humans and birds have been observed. The disease is of concern because most patients have become severely ill. Most of the human cases with H7N9 infection have reported recent exposure to live poultry or potentially contaminated environments, especially markets where live birds have been sold. This virus does not appear to be transmitted easily from person to person, and sustained human-to-human transmission has not been reported [32, 33].

“H7N9 influenza infection” was listed as a Category V Notifiable Infectious Disease starting from April 3, 2013, in Taiwan, and on April 24, three weeks later, Taiwan CDC announced that a 53-year-old man who had recently traveled to China is hospitalized in critical condition with a novel H7N9 infection; it is the first case of H7N9 detected outside of China [34, 35]. Taiwan CDC continued to follow up on 139 people who had had contact with the patient, and none of them was positive for the H7N9 virus. The Center for Research and Diagnostics of Taiwan CDC took another preparedness step by developing diagnostic test to detect this new virus and issued a technical briefing to the contracted virology laboratories for screening H7N9 infections. The materials are also available on the Taiwan CDC's website [36]. So far the source of the virus appears to be birds, especially poultry and the environment contaminated by the virus, and the risk of infections seems most concentrated in live-poultry markets. Though a few family clusters have been found, experts found no evidence to conclude that person-to-person transmission is occurring and that no sustained transmission has been found. However, it is noted that limited person-to-person transmission is demonstrated in China [37].

## 7. First Human H6N1 Infection

On May 20, 2013, the first human-infected case of H6N1 avian influenza in the world was reported from Taiwan. This novel avian-origin influenza A (H6N1) virus was confirmed by the National Influenza Center, Centers for Disease Control, Taiwan. The patient was sick with pneumonia and has already recovered. Avian H6 subtype of influenza viruses was first identified in turkeys in 1965 and makes up one of the most commonly recognized subtypes in domestic ducks in Southern China [38]. Previous studies indicated that the H6N1 virus is low pathogenic [39].

This H6N1 virus was identified at the time when Taiwan CDC was on heightened alert for H7N9 illnesses. A 20-year-old woman's novel flu infection first came to the attention of Taiwanese health authorities just 2 weeks after the region had identified its first H7N9 case in a patient who had recently traveled to China's outbreak area for work. The woman started having flu-like symptoms on May 5 including fever, cough, headache, and muscleache. She was hospitalized 3 days later after being diagnosed with mild pneumonia, and her condition quickly improved after she was treated with oseltamivir (Tamiflu). Tests on her respiratory specimen at the hospital indicated an unsubtypable influenza A virus, and the virus isolated from respiratory specimen of the case was then submitted to Taiwan CDC for further identification. After conducting whole genome sequencing, the Taiwan NIC identified the virus as a novel avian-origin influenza A H6N1 virus [40]. Each year, on average, approximately ten to twenty thousand specimens were tested. A total of 250,000 cumulative specimens have been tested, in which eighty thousand influenza viruses have been isolated [39]. Except this H6N1 strain and the imported H7N9 strain detected in 2013, only the H1 and H3 subtypes of influenza A viruses have been detected in humans in Taiwan.

The H6N1 virus is a low-pathogenicity virus that commonly circulates in domestic birds, and the virus isolated from the patient's respiratory sample closely resembles the virus in poultry. Based on sequences of the genes encoding hemagglutinin and neuraminidase, the source of infection was not identified yet. Sequence analyses showed that this human isolate was highly homologous to chicken H6N1 viruses in Taiwan and had been generated through interclade reassortment. Notably, the virus had a G228S substitution in the hemagglutinin protein, as in human-adapted H3 strains, which effectively raises the possibility that H6N1 might be transmitted to humans [41]. Sequencing data suggest that it is sensitive to the neuraminidase inhibitors oseltamivir and zanamivir. The epidemiology of low-pathogenicity viruses such as H6N1 across Asia is largely unknown. However, such viruses are likely to be endemic in the same areas for a long time. The surge of human infections with H7N9 viruses in a large geographic area in China would seem to support such opinion. This case reminds us that health authorities must keep a close eye on the viruses that are circulating in Taiwan.

## 8. Conclusion

Influenza pandemics, sometimes with tremendous mortality, can arise from changes to influenza viruses circulating back and forth around the world. Human influenza viruses are dynamic and complex involving multiple avian, swine, and human gene origins, reflecting a continuous and opportunistic ability of the virus to reinvent itself and reinfect populations, as for the newly reported one human case of H6N1 infection in Taiwan and the H10N8 that has killed 3 on Mainland China. However, both H6N1 and H10N8 viruses have no direct evidence link to the H7N9 strain.

Although the source of H7N9 virus infection in patients with confirmed cases who had had contact with animals cannot be verified, most of the scientists suspect that it is likely to be infected poultry. Sporadic H7N9 bird flu human infections have been reported in Southern China and are usually associated with exposure to poultry. So far, no evidence shows that the virus can be transmitted between people, but both animal-to-human and human-to-human routes of transmission are being investigated. The H1, H2, and H3 subtypes are the only subtypes in the past century that have established stable lineages in humans, raising the possibility that only these subtypes can infect humans. However, all subtypes of influenza A virus that become established in mammals emerge from nature reservoirs. Influenza is a global challenge, and the prevention and control of influenza require a commensurate global response. The best way to detect, monitor, and control the next pandemic is to build strong national and global surveillance, understand the epidemiology and risk factors for seasonal and pandemic influenza, and promote interventions, such as vaccine, antiviral drugs, and nonpharmaceutical interventions in different settings.

Further pandemics of influenza are inevitable, as the ultimate reservoirs of influenza A viruses in wild aquatic birds cannot be eradicated. The opportunities for exchanging viruses between species and for reassortment of their genes have increased as the populations of human, swine, and birds have increased. As we learn more about influenza viruses in wild-bird reservoirs and the genomics of virus required to be transmitted to intermediate hosts and ultimately among humans, we may be able to make predictions about which influenza viruses have pandemic potential.
